# 5-HTTLPR and use of antidepressants after colorectal cancer including a meta-analysis of 5-HTTLPR and depression after cancer

**DOI:** 10.1038/tp.2015.121

**Published:** 2015-09-01

**Authors:** N P Suppli, J D Bukh, T E Moffitt, A Caspi, C Johansen, V Albieri, A Tjønneland, L V Kessing, S O Dalton

**Affiliations:** 1 Unit of Survivorship, Danish Cancer Society Research Center, Copenhagen, Denmark; 2 Psychiatric Center Copenhagen, University of Copenhagen, Copenhagen, Denmark; 3 Department of Psychology and Neuroscience, Center for Genomic and Computational Biology, Duke University, Durham, NC, USA; 4 Department of Psychiatry and Behavioral Sciences, Center for Genomic and Computational Biology, Duke University, Durham, NC, USA; 5 Social, Genetic, and Developmental Psychiatry Research Center, Institute of Psychiatry, King's College London, London, UK; 6 Department of Oncology, Finsencentret, Rigshospitalet, Copenhagen, Denmark; 7 Unit of Statistics, Bioinformatics and Registry, Danish Cancer Society Research Center, Copenhagen, Denmark; 8 Unit of Diet, Genes, and Environment, Danish Cancer Society Research Center, Copenhagen, Denmark

## Abstract

The serotonin-transporter-linked polymorphic region (5-HTTLPR) is one of the most extensively investigated candidates to be involved in gene–environment interaction associated with depression. Nevertheless, the interaction remains controversial. In an original study, we tested the hypothesis that risk for use of antidepressants following a diagnosis of colorectal cancer is associated with bi- and triallelic genotypes of 5-HTTLPR. In addition, in an inclusive meta-analysis, we tested the hypothesis that depression following a diagnosis of cancer is associated with biallelic 5-HTTLPR genotype. We created an exposed-only cohort of 849 colorectal cancer patients from the Danish Diet, Cancer and Health cohort study. The hypothesized association was investigated with Cox regression models and competing risk analyses. Five studies comprising a total of 1484 cancer patients were included in the meta-analysis. Nationwide registries provided information on dates of diagnosis of colorectal cancer and use of antidepressants. Unadjusted odds ratios of depression according to the biallelic 5-HTTLPR genotype were included in the meta-analysis. 5-HTTLPR genotypes were not associated with use of antidepressants after colorectal cancer. Estimated hazard ratios ranged 0.92–1.08, and we observed no statistically significant associations across biallelic and triallelic genotypes in crude as well as adjusted models. The meta-analysis showed no statistically significant associations of 5-HTTLPR biallelic genotype with depression after cancer. Our findings in an original study and a meta-analysis do not support the hypothesis of an association between the 5-HTTLPR genotype and depression after cancer.

## Introduction

The serotonin-transporter-linked polymorphic region (5-HTTLPR) is one of the first, most extensively investigated and promising candidates to be involved in gene–environment interaction associated with a psychiatric disorder.^1^ Nonetheless, after more than 80 individual studies and four meta-analyses^2, 3, 4, 5^ there is still no consensus if or under which conditions 5-HTTLPR genotype affects vulnerability to depression when a person faces a severely stressful life event.^6^

The two most recent meta-analyses provide support for the hypothesized interaction.^4, 5^ However, a large number of well-conducted studies exist that do not find this. This indicates that the influence of 5-HTTLPR and stress on risk for depression is complex.^5^ It might exist only in certain windows of vulnerability over the life course or in specific types of stressful life events; it might be modified by sex or be found only for certain subtypes or degrees of severity of depression. One lead is to focus further research on interactions between 5-HTTLPR and severe medical conditions for which the meta-analyses found substantial support for an effect on depression risk.^4, 5^ Moreover, it is suggested that studies based on objective measurement of both stress and depression generate the most consistent support for the hypothesis.^5^

With the present study we aim to contribute to the understanding of the 5-HTTLPR × stress interaction on depression by adding a large, Danish, register-based cohort study to the promising area of 5-HTTLPR × medical-illness studies.

We had the opportunity to use diagnosis of colorectal cancer, known to increase risk for depression, as the severely stressful life event.^7, 8, 9^ In addition, we applied use of antidepressants as a measure of pharmacologically treated depression. In European countries comparable to Denmark ~50% of patients make treatment contact in the year of onset of mood disorders^10^ and, in Denmark, pharmacological treatment is the primary recommended treatment for moderate and severe depressions.^11^ As antidepressants are available on prescription only, treatment with antidepressants is an indication of a patient with functional impairment recognized and pharmacologically treated by a physician. Although antidepressants are used to treat other conditions, depression is the indication of 71–86% of prescribed antidepressants, with anxiety, also hypothesized to be affected by 5-HTTLPR × stress interaction, being the second most common indication.^12, 13, 14, 15^

Using exact dates of diagnosis of colorectal cancer and redeemed prescriptions of antidepressants, we tested the hypothesis that risk for use of antidepressants following diagnosis of colorectal cancer is associated with bi- and triallelic genotypes of 5-HTTLPR. This constitutes a unique study design that avoids biases and errors associated with recall of subjective and past events by combining clinical measures of stress and depression, including their temporal order. Finally, we performed a meta-analysis of studies investigating the association between 5-HTTLPR and depression in cancer patients.

## Materials and methods

### Study design

The present study is an exposed-only cohort study of all 874 participants from the Danish Diet, Cancer, and Health cohort study diagnosed with a first primary colorectal cancer 1998–2009. Using the unique personal identification number assigned to all residents in Denmark, we were able to identify exposure, outcome and relevant covariates from nationwide registers. Participants were followed from the date of diagnosis of colorectal cancer until pharmacologically treated depression, operationalized as first redeemed prescription of an antidepressant, or any of the following censoring events: emigration, diagnosis of a new primary cancer (except non-melanoma skin cancer), hospital contact with a diagnosis of organic mental disorder, mental or behavioral disorder due to use of a psychoactive substance, schizophrenia, schizotypal or delusional disorder, or manic or bipolar episodes (International Classification of Disease 8 (ICD-8): 290–295.99, 296.19, 296.39, 303.00–304.99 and ICD-10: F00–F31), death, or 31 December 2011, whichever came first.

Persons who emigrated before diagnosis of colorectal cancer were excluded (*n*=3). Further, to ensure that use of antidepressants was not related to the aforementioned psychiatric disorders, we excluded persons diagnosed with any of these before diagnosis of colorectal cancer (*n*=22). After exclusions, a study population of 849 colorectal cancer patients was available for genotyping.

### The Diet, Cancer and Health cohort study

Between December 1993 and May 1997 all 160 725 men and women aged 50–64, born in Denmark, without any history of cancer, and living in Copenhagen or Aarhus areas, were invited to participate in the cohort. A total of 57 053 participants were recruited. Comprehensive baseline information as well as blood samples with extracted DNA exist for all participants. The Diet, Cancer and Health cohort study has been approved by the Ethical Committees on Human Studies in Copenhagen and Aarhus (KF 01-345/93), and the Danish Data Protection Agency (2013-41-2043) and informed consent was given from all participants at inclusion. A complete description of the cohort is provided elsewhere.^16^

### Genotyping of the serotonin-transporter-linked polymorphic region (5-HTTLPR)

The 5-HTTLPR is a polymorphic tandem repeat in the 5' promoter region of the gene encoding the serotonin transporter (SLC6A4) with two common alleles: a long (L) allele and a less transcriptionally efficient short (S) allele.^17^ Further, an A→G single-nucleotide polymorphism (rs25531) has been identified within the long allele of the 5-HTTLPR, yielding a third allele, L_G_, which shows a transcriptional activity similar to that of the S allele.^18^

Genotyping was carried out using TaqMan chemistry. Primers used for both the L/S and the rs25531 assays were pF: 5′-GCAACCTCCCAGCAACTCCCTGTA-3′ and pR: 5′-GAGGTGCAGGGGGATGCTGGAA-3′. Probe sequences used for genotyping of the L/S allele status were L/S long allele probe: 6FAM-5′-TGCAGCCCCCCCAGCATCTCCC-3′-MGB and L/S control probe: VIC-5′-TCCCCCCCTTCACCCCTCGCGGCATCC-3′-MGB. Probe sequences used for genotyping of rs25531 allele status were A probe: 6FAM-5′-CCCCCCTGCACCCCCAGCATCCC-3′-MGB and G probe: VIC-5′-CCCCTGCACCCCCGGCATCCCC-MGB. Separate 384-well plates were prepared for genotyping of L/S and rs25531 allele status. The diluted primers and probes were mixed with ABI TaqMan genotyping master mix, ethylenediaminetetraacetic acid, dimethyl sulfoxide and PCR grade water. Plates were then thermally cycled. Subsequently, Vic versus Fam fluorescence was plotted and the clusters analyzed with the KBioscience Kraken tool (Hertfordshire, UK) to generate genotyping results.

The biallelic genotype was grouped as LL, SL and SS. The triallelic genotype was grouped according to the hypothesized functional gene-expression activity (High: L_A_L_A_; Medium: L_G_L_A_, SL_A_; Low: SS, SL_G_, L_G_L_G_).

Results of genotyping allowed us to determine the biallelic genotype in 806 (95%) patients and the triallelic genotype in 793 (93%) patients (Table 1). Both biallelic (*X*^2^=2.28, *P*=0.13) and triallelic (*X*^2^=3.51, *P*=0.32) genotypes were in Hardy–Weinberg equilibrium.

### Information from registers

Information on all cancers diagnosed during 1943–2011 was identified in the Danish Cancer Registry, which is known for its completeness and high validity.^19^ Date of cancer diagnosis was defined as date of first registered hospital contact related to the relevant cancer.

Since 1995 all prescription drugs redeemed at Danish pharmacies have been registered in the Danish National Prescription Registry.^20^ In Denmark, antidepressants are available by physicians' prescription only, and we obtained date of issue for all antidepressants (Anatomical Therapeutic Group N06A) redeemed in 1995–2011 by study participants.

From the Central Population Register, we obtained dates of migration and death for all study participants. From the Danish Psychiatric Central Register, we obtained dates and diagnoses for all contacts with psychiatric hospitals during 1969–2011. The register holds information on all inpatient admissions since 1969 as well as all outpatient contacts since 1995.^21^

Since 1978 the Danish Patient Registry has registered all patients admitted to Danish hospitals including all outpatient contacts since 1995. We obtained dates and diagnoses from all admissions for all study participants in 1978–2011. On the basis of these diagnoses the Charlson comorbidity index score at the time of diagnosis of colorectal cancer was calculated for all study participants.^22^

From the Integrated Database for Labor Market Research, we obtained information on the highest level of attained education and cohabitation status for all study participants in the year of diagnosis of colorectal cancer.^23^

Characteristics of the colorectal cancer patients according to bi- or triallelic genotypes are shown in Table 1, with no statistically significant differences in baseline characteristics between groups.

### Statistical analyses

Incidence density rates of use of antidepressants were calculated annually 3 years before diagnosis of colorectal cancer and 5 years after diagnosis. The calculations were based on number of observed new users of antidepressants in a given 1-year time period divided by the total number of person-days during that specific period.

We tested whether characteristics at diagnosis of colorectal cancer and follow-up times were distributed randomly across bi- and triallelic genotypes using analysis of variance for continuous variables and *X*^2^-tests for categorical variables.

The associations between 5-HTTLPR bi- and triallelic genotypes and use of antidepressants after colorectal cancer were evaluated with Cox regression modeling. After assessment of linearity, the variables ‘age at diagnosis of colorectal cancer' and ‘calendar year at diagnosis of colorectal cancer' were included as continuous variables. We visually assessed plots of log minus log of the survival density function versus log of follow-up time. No variables were considered to violate the assumption of proportionality. We present results of a crude model as well as one multivariable model adjusting for age, calendar year and sex, and another multivariable model additionally adjusting for educational level, comorbidity and cohabitation status. In order to study the special case of incident use of antidepressants after colorectal cancer, we further used the above models to do subanalyses including only patients who had never had a hospital contact with unipolar depression (ICD-8: 296.09, 296.29 or ICD-10: F32-F33.9) and who did not redeem any antidepressants during the 3 years before diagnosis of cancer.

Cumulative incidence functions were estimated on the basis of a Cox regression model for competing-risks survival data with death as a competing risk according to Rosthøj *et al.*^24^ On the basis of the cumulative incidence functions, a plot of cumulative use of antidepressants after diagnosis of colorectal cancer was plotted as a function of time since diagnosis, stratified by genotype. Test of difference between groups was carried out with Gray's test.

For the purpose of the meta-analysis, we identified previous studies in PubMed and EMBASE using the search terms ‘5-HTTLPR OR serotonin transporter OR sert, AND depression OR depressed, AND cancer OR neoplasm OR malignant OR tumor OR tumor'. This search gave 55 hits in PubMed and 115 initial hits in EMBASE. Six studies were identified as relevant through screening of titles and abstracts followed by reading of full texts.^25, 26, 27, 28, 29, 30^ We wanted separate meta-analytic risk estimates for the SS genotype and SL genotype, with LL genotype as comparison. One study reported separate odds ratios in the desired form,^22^ two studies reported enough data to calculate odds ratios^19, 20^ and authors from one study group were able to provide relevant data on request.^17^ Two research groups were not able to provide the requested data, and these results were thus not included in the analyses.^18, 21^ For the purpose of meta-analysis, odds ratios were calculated for the present study using numbers of users and non-users of antidepressants in each biallelic genotype group. A meta-analysis using random-effects models was computed to investigate an overall association between 5-HTTLPR and depression in cancer patients. All odds ratios included in the meta-analysis were unadjusted and inverse variance weighting was used for pooling.

Cox regression and competing risk analyses were performed by author NPS with the PHREG procedure and the CumInc macro^24^ in SAS version 9.3 (SAS Institute, Cary, NC, USA). Gray's test for differences between groups in competing risk analyses was conducted by author VA with the package ‘cmprsk' and meta-analysis using ‘meta' and ‘metafor' in R version 3.1.2 (www.R-project.org).

## Results

**Figure 1:**
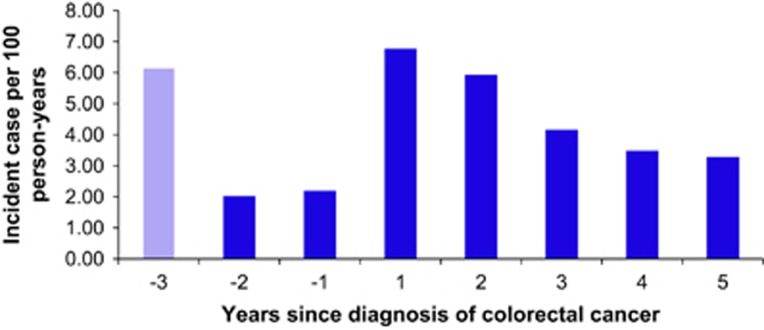
Incident users of antidepressants before and after diagnosis of cancer among all 849 colorectal cancer patients diagnosed in 1998–2009. To exclude prevalent users of antidepressants the third year before cancer (−3) was used as a wash-in period. The second year before cancer (−2) is thus the first year in which we observe incident use of antidepressants. The incidence was calculated as number of new users divided by the total number of days (converted to years) contributed to each time period. All individuals were followed for first redeemed prescription of an antidepressant and, until emigration, new diagnosis of cancer (except non-melanoma skin cancer), diagnosis of a psychiatric disorders to which depression could be considered secondary (ICD-8: 290–295.99, 296.19, 296.39, 303.00–304.99 and ICD-10: F00–F31), death or 5 years after diagnosis of colorectal cancer, whichever came first. ICD-8, International Classification of Disease 8.

Colorectal cancer increased the risk for use of antidepressants. On the basis of 15 new users of antidepressants in the year before diagnosis of colorectal cancer and 41 new users in the first year after diagnosis, we estimate that incident use of antidepressants increased from 2.2 per 100 person-years in the year before diagnosis to 6.8 per 100 person-years in the first year after diagnosis (Figure 1).

Among the 806 colorectal cancer patients in whom biallelic genotype was successfully determined, the mean follow-up time was 4 years (Table 1). During follow-up, six patients (1%) had a hospital contact with depression (data not shown), and 180 patients (22%) were registered as users of antidepressants (Table 2). Further, among these 806 patients, 385 (48%) died before 31 December 2011 and 220 (27%) had death as first event in the Cox analyses, with no differences between biallelic genotype groups (*P*=0.54). We found no significant differences in baseline characteristics between genotype groups (Table 1).

Genotype was not associated with use of antidepressants after colorectal cancer. This was true whether we examined biallelic or triallelic genotypes and whether we estimated crude or adjusted models (Table 2). Estimated hazard ratios for use of antidepressants after diagnosis of colorectal cancer ranged from 0.92 to 1.08, and no statistically significant effects were observed (Table 2). Neither did subanalyses of colorectal cancer patients who had never had a hospital contact with unipolar depression and who did not redeem antidepressants during the 3 years before diagnosis of cancer show any significant associations between bi- or triallelic genotypes and use of antidepressants after cancer (Supplementary Information, Supplementary Table).

**Figure 2:**
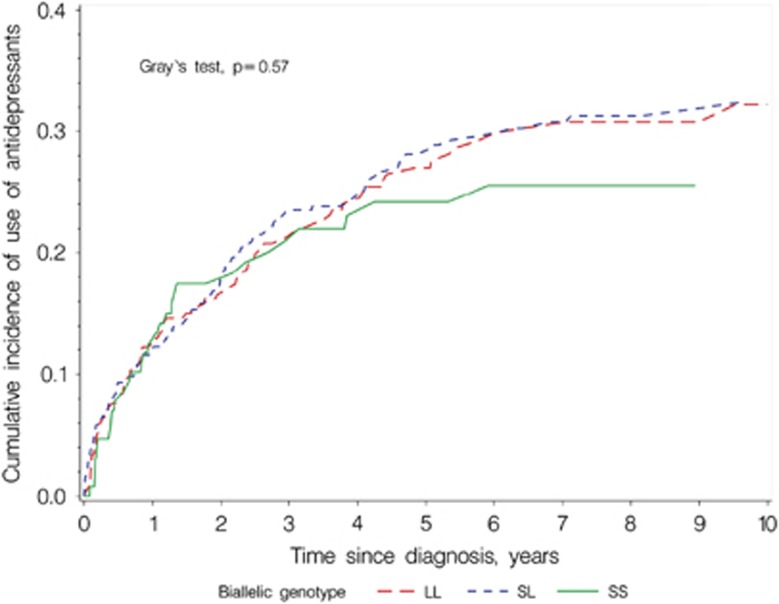
Estimated cumulative incidence functions of use of antidepressants according to 5-HTTLPR biallelic genotype in 806 colorectal cancer patients. 5-HTTLPR, serotonin-transporter-linked polymorphic region.

In the competing risk-analysis we found no statistical difference between biallelic genotypes in the estimated cumulative use of antidepressants after diagnosis of colorectal cancer (Figure 2).

**Figure 3:**
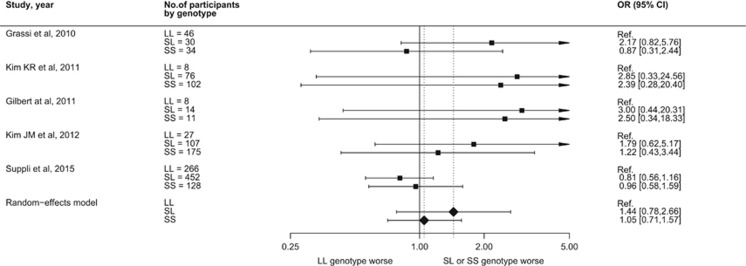
Forest plot showing risk of depression in cancer patients associated with 5-HTTLPR SS and SL genotypes compared with the LL genotype. 5-HTTLPR, serotonin-transporter-linked polymorphic region.

On the basis of four previous studies including a total of 33 head and neck cancer patients and 642 breast cancer patients plus 806 colorectal cancer patients from the present study, we did not find that the SL genotype (meta-analytic risk estimate 1.44, 95% CI 0.78–2.65) or SS genotype (meta-analytic risk estimate 1.05, 95% CI 0.71–1.57) was associated with statistically significant increased odds of depression after diagnosis of cancer (Figure 3).

## Discussion

In the reported register-based cohort study and meta-analysis we found no significant associations of use of antidepressants or depression with 5-HTTLPR in cancer patients. Among participants diagnosed with colorectal cancer, the 5-HTTLPR genotype was not associated with use of antidepressants, our clinical measure of depression recognized and pharmacologically treated by a physician. Result from the meta-analysis did not support a general association of 5-HTTLPR with depression after cancer.

The present study is characterized by several strengths. First, rather than investigating gene × environment interactions by focusing on statistical interactions, which are vulnerable to many artifacts,^31^ we explicitly studied genetic sensitivity to the environment by focusing on a group of individuals exposed to a relatively uniform environmental exposure and testing whether genotype influenced risk for the clinical outcome use of antidepressants. Even though colorectal cancer covers a wide spectrum of disease severities and treatment intensities, by using diagnosis of colorectal cancer as a stressor we made certain that all participants were exposed to stress that increases risk for depression.^7, 8^ Accordingly, use of antidepressants was substantially increased following the diagnosis of colorectal cancer compared with before. Second, use of national registries allowed unbiased assessment of colorectal cancer, use of antidepressants and their temporal order. Third, in contrast to prior studies that were all restricted to assessments of point prevalence of depression, our design enabled us to continuously follow cancer patients from the date of diagnosis up to 14 years after diagnosis.

The results of the present study should, however, be evaluated in light of limitations. First, although our clinical outcome—use of antidepressants—in fundamental aspects allowed an advantageous design, drawbacks related to misclassification are also inherent. Depression is indeed the indication for the vast majority of prescribed antidepressants, with anxiety being the second most common.^12, 13, 14^ Nevertheless, conditions such as hot flushes and pain are also used as indications for some antidepressants. In addition, not all persons with depression are treated with antidepressants. Thus, we caution that our results are on the basis of the assumption that 5-HTTLPR would affect incidence of depression, while at the same time not affecting the probability of detection and treatment of individual depressions. Further, we also assume that incidence, detection and treatment with antidepressants of conditions unrelated to depression are independent of 5-HTTLPR. In addition, our outcome did not allow us to discriminate between subtypes of depression, which might also influence the investigated gene–environment interaction.^5^ Second, as colorectal cancer is depressionogenic we assume that such a diagnosis is distressing; however, we did not measure subjective perceptions of stress. As such, we were not able to capture wide individual differences in the perceived stress associated with receiving a colorectal cancer diagnosis, and we necessarily assumed that the diagnosis was equally distressing to all individuals. This is a simplification as persons will react differently to being diagnosed with cancer, and colorectal cancer covers a wide spectrum of disease severities and treatment intensities. Third, we were not able to determine the bi- and triallelic genotypes in all participants. However, the genotypes were in Hardy–Weinberg equilibrium, suggesting that unsuccessful genotyping was non-differential. The size of the study population was predefined and therefore power calculations had no purpose when planning our study. The narrow confidence intervals for hazard ratios reported in Table 2 and the Supplementary Table illustrate sufficient statistical power supporting the idea that *post hoc* power calculations would be inappropriate.^32^

The present study is the seventh to investigate the hypothesis that 5-HTTLPR modifies the association between cancer and depression, doubling the total number of cancer patients evaluated.^25, 26, 27, 28, 29, 30^ All previous studies have been smaller (*n*=33–309), so much so that in some even large observed differences in depression between 5-HTTLPR genotype groups have not reached statistical significance. Only one of the previous studies about cancer patients was included in previous meta-analyses of the link between 5-HTTLPR, stress and depression.^17^ The two studies that we were not able to include in our meta-analysis comprise a total of 133 cancer patients, and both studies reported nonsignificantly higher risk of depression in cancer patients with a least one s allele.^26, 29^ As our meta-analysis is based on few studies consisting almost exclusively of breast and colorectal cancer patients, we advise that it is interpreted with caution. In addition, factors not accounted for, such as sex, age, ethnicity, timing of assessment of depression and type of depression measure, could affect the hypothesized association.

In contrast to large meta-analyses that have provided substantial evidence of 5-HTTLPR being associated with vulnerability for depression after stressful life events, our results indicate that the association is not present in populations of cancer patients. More studies based on larger populations as well as studies of patients with other types of cancer than colorectal and breast should be conducted before a definitive conclusion is warranted. However, currently, we do not find evidence supporting that the 5-HTTLPR genotype affects risk for depression in cancer patients.

## Figures and Tables

**Table 1:** Characteristics of participants diagnosed with colorectal cancer according to 5-HTTLPR biallelic and triallelic genotype

	*Biallelic genotype*				*Triallelic genotype*			
	*LL (*n=*266)*	*SL (*n=*412)*	*SS (*n=*128)*	P*-value*	*L*_*A*_*L_A_ (*n=206)	*L*_*A*_*L*_*G*_*, SLA* *(*n=*399)*	*L*_*G*_*L*_*G*_*, SL*_*G*_*, SS (*n=*188)*	P*-value*
*Age at diagnosis (years)*								
Mean (5%, 95%)	66.4 (57,76)	66.3 (57, 76)	66.4 (57, 75)	0.95	66.2 (58, 76)	66.5 (57,76)	66.2 (57,75)	0.77
Sex, number of females (%)	114 (43)	171 (42)	58 (45)	0.74	86 (42)	167 (42)	83 (44)	0.85
								
*Educational level, n (%)*								
Basic	49 (18)	72 (17)	29 (23)		35 (17)	71 (18)	41 (22)	
Vocational	152 (57)	219 (53)	67 (52)	0.51	116 (56)	217 (54)	98 (52)	0.79
High	61 (23)	118 (29)	31 (24)		52 (25)	108 (27)	48 (26)	
Unknown	4 (2)	3 (1)	1 (1)		3 (1)	3 (1)	1 (1)	
								
*Cohabitation status at diagnosis, n (%)*								
Living alone	84 (32)	116 (28)	37 (29)	0.63	65 (32)	119 (30)	49 (26)	0.47
Living with a partner	182 (68)	296 (72)	91 (71)		141 (68)	280 (70)	139 (74)	
								
*Charlson comorbidity index score, n (%)*								
0	177 (67)	268 (65)	89 (70)	0.67	135 (66)	266 (66)	126 (67)	0.80
1	51 (19)	71 (17)	21 (16)		42 (20)	67 (17)	32 (17)	
⩾2	38 (14)	73 (18)	18 (14)		29 (14)	66 (16)	30 (16)	
								
*Year of diagnosis (calendar year)*								
Mean (5%, 95%)	2003.9 (1998, 2009)	2004.1 (1998, 2009)	2003.5 (1998, 2009)	0.25	2003.9 (1999, 2009)	2004.1 (1998, 2009)	2003.5 (1998, 2009)	0.08
								
*Follow-up time (years)*								
Mean (5%, 95%)	4.1 (0.1, 10.8)	4.0 (0.1, 11.2)	4.2 (0.1, 12.0)	0.85	4.2 (0.1, 10.8)	4.0 (0.1, 10.9)	4.1 (0.1, 12.0)	0.89

Abbreviations: ANOVA, analysis of variance; 5-HTTLPR, serotonin-transporter-linked polymorphic region.

Tests for differences between allele groups were done with ANOVA for continuous variables and *X*^2^-tests for categorical variables.

**Table 2:** Cox regression analyses of risk for use of antidepressants according to 5-HTTLPR biallelic and triallelic genotypes

*Serotonin-transporter-functional genotype*	*Person-years*	*Users of antidepressants*, n	*Unadjusted model*		*Model 1*		*Model 2*	
			*HR*	*(95% CI)*	*HR*	*(95% CI)*	*HR*	*(95% CI)*
*Biallelic genotype (*n=*806)*								
LL	1084	62	1	—	1	—	1	—
SL	1644	89	0.93	0.7–1.3	0.92	0.7–1.3	0.92	0.7–1.3
SS	536	29	0.96	0.7–1.5	0.95	0.6–1.5	1.03	0.7–1.6
Total	3264	180						
								
*Triallelic genotype (*n=*793)*								
L_A_L_A_	856	48	1	—	1	—	1	—
L_A_L_G_, SL_A_,	1599	87	0.96	0.7–1.4	0.95	0.7–1.4	0.98	0.7–1.4
L_G_L_G_, SL_G_, SS	768	42	1.00	0.7–1.5	1.01	0.7–1.5	1.08	0.7–1.6
Total	3223	177						

Abbreviations: CI, confidence interval; HR, hazard ratio; 5-HTTLPR, serotonin-transporter-linked polymorphic region.

Model 1: adjusted for age at diagnosis, calendar year at diagnosis and sex.

Model 2: adjusted for age at diagnosis, calendar year at diagnosis, sex, educational level at diagnosis (basic, vocational, high), Charlson Comorbidity Index score at diagnosis (0, 1, +2) and cohabitation status at diagnosis (cohabiting or single).
