# Supplementary material for: 5-HTTLPR and use of antidepressants after colorectal cancer including a meta-analysis of 5-HTTLPR and depression after cancer
Source: Transl Psychiatry. 2015 Sep 1;5(9):e631–. doi: 10.1038/tp.2015.121 (PMC5068816; doi:10.1038/tp.2015.121)
Supplement: Supplementary Table [file tp2015121x1.doc]

# Supplementary Information

| Table 3. Cox regression analyses of risk for incident use of antidepressants according to 5-HTTLPR biallelic and triallelic genotype | | | | | | | | | | | | |
| --- | --- | --- | --- | --- | --- | --- | --- | --- | --- | --- | --- | --- |
| Serotonin transporter  functional genotype | | Person years | Users of antidepressants, n |  | Unadjusted model | |  | Model 1 | |  | Model 2 | |
|  | |  |  |  | HR | (95% CI) |  | HR | (95% CI) |  | HR | (95% CI) |
| Biallelic genotype (n=766) | |  |  |  |  |  |  |  |  |  |  |  |
| LL | | 1040 | 55 |  | 1 | - |  | 1 | - |  | 1 | - |
| SL | | 1583 | 78 |  | 0.91 | (0.7-1.3) |  | 0.91 | (0.6-1.3) |  | 0.90 | (0.6-1.3) |
| SS | | 533 | 23 |  | 0.84 | (0.5-1.4) |  | 0.83 | (0.5-1.4) |  | 0.90 | (0.6-1.5) |
| Total | | 3156 | 156 |  |  |  |  |  |  |  |  |  |
| Triallelic genotype (n=753) | |  |  |  |  |  |  |  |  |  |  |  |
| L_A_L_A_ | | 827 | 41 |  | 1 | - |  | 1 | - |  | 1 | - |
| L_A_L_G_, SL_A_, | | 1526 | 76 |  | 0.98 | (0.7-1.4) |  | 0.97 | (0.7-1.4) |  | 0.97 | (0.6-1.4) |
| L_G_L_G_, SL_G_, SS | | 763 | 36 |  | 0.98 | (0.6-1.5) |  | 1.00 | (0.6-1.6) |  | 0.99 | (0.6-1.6) |
| Total | | 3116 | 153 |  |  |  |  |  |  |  |  |  |
| Model 1:  Model 2: | adjusted for age at diagnosis, calendar year at diagnosis, and sex  adjusted for age at diagnosis, calendar year at diagnosis, sex, educational level at diagnosis (basic, vocational, high), Charlson Comorbidity Index score at diagnosis (0, 1, +2), and cohabitation status at diagnosis (cohabiting or single) | | | | | | | | | | | |
